# Bibliographical

**Published:** 1894-01

**Authors:** 


### Bibliographical.

A Practical Treatise on Mechanical Dentistry, by Joseph Richardson, M.D., D.D.S. Sixth Edition. Revised and edited by Geo. W. Warren, D.D.S., with six hundred illustrations.

The fifth edition of this work was issued September, 1888. The demand for the sixth edition within five years of that time is a very clear indication not only of the popularity of the work but of its intrinsic merit.

It has, since the issue of the first edition, stood as a treatise upon Prosthetic Dentistry for the student and the younger practitioner at the head of the list of works of this character, and not only for these has it been valuable but for the general and older practitioner as well, as is clearly proven by the frequent reference to it by every practitioner.

The author of the work having passed away since the fifth edition was issued, the preparation of this necessarily fell into other hands, and it is hard to conceive that it could have been better done than it has by Dr. Warren, the present editor, who says, in a note attached: "In preparing the sixth edition of this work, the editor's first effort has been to make it pre-eminently practical as a text-book for students and a guide for young practitioners. Useless methods and obsolete theories have been eliminated; much of the text has been re-written, notably the section on Crown and Bridge-Work. It has also been newly illustrated, bringing the treatise fully up to the time of its publication."

The rapid strides made and continually being made in Prosthetic Dentistry render it difficult to prepare a work of this kind that shall faithfully represent the progress in this direction for more than two or three years. Notwithstanding the basal principles upon which this department of dental practice is based, are quite well established and doubtless will remain, with some modification perhaps, in the long future, but in details, changes are being constantly made; this pertains not only to manipulation and progress, but to materials and their application as well. In this latter particular Dr. Warren has succeeded admirably, and the volume as presented is quite up to the present standing of Prosthetic Dentistry.

A large addition has been made to the illustrations, which adds much to the value of the work. These are very well executed, and add much to the facility of comprehending the text, though it is clear and concise. So large a number of new illustrations have been added that it might almost be said to be newly illustrated throughout.

The department on Crown and Bridge-Work is so fully and so well presented that it would seem almost to render unnecessary any other work on this subject, occupying over two hundred pages of the work.

This edition, as was the case with the others, cannot be too strongly commended to the attention of every member of our profession who desires to keep up with its progress.

The work is published by P. Blakiston, Son & Co., of Philadelphia.

---

Diseases and Injuries of the Teeth, Including Pathology and Treatment. A Manual of Practical Dentistry for Students and Practitioners, by Morton Smale, M.R.C.S. L.S.A., L.D.S., and J. F. Colyer, L.R.C.P., M.R.C.S., L.D.S. Published by Longmans, Green & Co., London and New York.

The above work has just come to hand. Opportunity has not yet been afforded to give it sufficient examination to speak definitely of its merits. Judging from the reputation and standing of the authors and a hasty glance at its contents, we are favorably impressed with the work; we will, however, have more to say upon it after a fuller examination.

